# Supplementary material for: An Easy-to-Implement Risk Score for Targeted Hepatitis C Virus Testing in the General Population
Source: Microbiol Spectr. 2022 Mar 31;10(2):e02286-21. doi: 10.1128/spectrum.02286-21 (PMC9045242; doi:10.1128/spectrum.02286-21)
Supplement: SUPPLEMENTAL FILE 1 — Supplemental material. Download SPECTRUM02286-21_Supp_1_seq3.pdf, PDF file, 0.1 MB [file spectrum02286-21_supp_1_seq3.pdf]

# An easy-to-implement risk score for targeted hepatitis C testing in the general population

## Supplementary material

## Questionnaire for HCV risk assessment

| | Risk of exposure | YES | NO |
|---|---|---|---|
| 1 | Have you ever had sexual intercourse without condom protection, which could represent a risk for acquiring HIV / AIDS in the last 30 years, unless you are with a close partner or always use condoms? | | |
| 2 | **Have you ever had a partner with HIV infection?** | | |
| 3 | **Are you a man who has ever had sex with men?** | | |
| 4 | **Had you received any blood or blood product transfusion before 1985?** | | |
| 5 | **Have you ever used any illicit or recreational intravenous drugs?** | | |
| 6 | **Do you think you could have acquired HIV or HCV infection for any reason?** | | |
| | **Have you ever had any of the diseases below?** | **YES** | **NO** |
| 7 | **Sexually transmitted infection (syphilis, gonorrhea, urethritis, genital herpes…)** | | |
| 8 | **Lymphoma** | | |
| 9 | **Cervical or anal cancer, or dysplasia** | | |
| 10 | Herpes Zoster | | |
| 11 | **Hepatitis B/C or unexplained liver disease** | | |
| 12 | Mononucleosis-like syndrome | | |
| 13 | **Thrombocytopenia/unexplained leucopenia** | | |
| 14 | Seborrheic dermatitis | | |
| 15 | Unknown origin/unexplained fever | | |
| 16 | Repeated oral or vaginal candidiasis without antibiotic use | | |
| 17 | Oral hairy leukoplakia | | |
| 18 | Unexplained prolonged (> 3 months) diarrhea | | |
| 19 | Unexplained weight loss | | |
| 20 | ***Mycobacterium tuberculosis* disease** | | |
| 21 | Pneumonia | | |

*All responses were considered a risk for HIV infection. Questions 2, 3, 4, 5, 6, 7, 8, 9, 11, 13, and 20 were considered a risk for HCV infection.

**Table S1. Comparison of the four primary care centers participating in the study.**

| PRIMARY CARE CENTERS | Mar Báltico | Canal de Panamá | García Noblejas | Aquitania |
|---|---|---|---|---|
| **Total staff, n** | 86 | 37 | 44 | 21 |
| **Daily attended patients (18-70 years-old)**, n | 1225 | 665 | 560 | 455 |
| **Study period (Intervention)** | 18/11/2016 - 03/02/2017 | 20/04/2017 - 14/06/2017 | 08/02/2017 - 16/03/2017 | 14/06/2017 - 18/09/2017 |
| **Days with intervention**, n | 52 | 40 | 27 | 68 |
| **Estimate of attended patients aged 18-70 on the study period** | 63700 | 26600 | 15120 | 30940 |
| **Age of attended population**, % | | | | |
| <15 years-old | 13.3 | 13.5 | 13.8 | 17.2 |
| 15-64 years-old | 57.1 | 56.8 | 60.1 | 62.1 |
| 65-74 years-old | 13.4 | 12.2 | 9.1 | 7.3 |
| ≥75 years-old | 16.2 | 17.4 | 17.0 | 13.4 |
| **Gender of attended population**, female % | 58.1 | 59.7 | 56.9 | 56.7 |

| | | | | |
|---|---|---|---|---|
| **Participants included in the study** | 1998 | 1994 | 1999 | 2000 |
| **Female gender**, % | 63.9 | 66.5 | 66.4 | 66.9 |
| **Age**, median (IQR) | 41 (31-52) | 42 (33-54) | 45 (32-54) | 45 (34-53) |
| **Origin**, % | | | | |
| Spain | 75.0 | 73.7 | 74.3 | 78.9 |
| Latin America | 16.4 | 15.8 | 16.5 | 12.7 |
| Africa | 0.6 | 1.6 | 1.1 | 1.1 |
| Eastern Europe | 2.1 | 2.7 | 2.1 | 2.2 |
| Western-Central Europe | 4.1 | 3.1 | 3.2 | 2.2 |
| Other | 1.9 | 3.1 | 2.6 | 2.9 |
| **Educational level**, % | | | | |
| None / Primary | 20.5 | 16.6 | 32.2 | 33.1 |
| Secondary | 42.5 | 37.9 | 40.8 | 38.2 |
| University | 34.3 | 44.3 | 25.2 | 27.0 |
| **Rapid HCV tests performed**, n | 1145 | 1126 | 1252 | 1194 |

**Table S2. Distribution of HCV test results for each total score obtained**

| | HCV test result | | |
|---|---|---|---|
| Score | Negative, n (%) | Positive, n (%) | Total, n |
| 0 | 1,140 (99.91) | 1 (0.09) | 1,141 |
| 1 | 744 (99.33) | 5 (0.67) | 749 |
| 2 | 147 (98.66) | 2 (1.34) | 149 |
| 3 | 134 (98.53) | 2 (1.47) | 136 |
| 4 | 83 (92.22) | 7 (7.78) | 90 |
| 5 | 15 (78.95) | 4 (21.05) | 19 |
| 6 | 6 (75.00) | 2 (25.00) | 8 |
| 7 | 3 (50.00) | 6 (50.00) | 6 |
| 8 | 0 (0.00) | 4 (100.00) | 4 |
| Total | 2,272 (98.7) | 30 (1.30) | 2,302 |

Abbreviations: HCV, hepatitis C virus

**Table S3. Detailed report of sensitivity and specificity for participants < 50 years**

| Cut-off point | Sensitivity | Specificity | LR+ | LR- |
|---|---|---|---|---|
| (≥ 0) | 100 | 0.0 | 1.000 | |
| (≥ 1) | 100 | 15.90 | 1.189 | 0.000 |
| (≥ 2) | 90.0 | 51.05 | 1.839 | 0.196 |
| (≥ 3) | 80.0 | 79.40 | 3.884 | 0.252 |
| (≥ 4) | 45.0 | 91.72 | 5.432 | 0.599 |
| (≥ 5) | 30.0 | 97.0 | 9.999 | 0.722 |
| (≥ 6) | 20.0 | 98.84 | 17.177 | 0.809 |
| (≥ 7) | 20.0 | 99.73 | 74.433 | 0.802 |
| (≥ 8) | 15.0 | 10.0 | | 0.850 |
| (> 8) | 0.0 | 10.0 | | 1.000 |

Abbreviations: LR+, positive likelihood ratio; LR-, negative likelihood ratio
